# Informational rescaling of PCA maps with application to genetic distance

**DOI:** 10.1016/j.csbj.2024.11.042

**Published:** 2024-12-11

**Authors:** Nassim Nicholas Taleb, Pierre Zalloua, Khaled Elbassioni, Haralampos Hatzikirou, Andreas Henschel, Daniel E. Platt

**Affiliations:** a Risk Engineering, School of Engineering, New York, USA; b Maroun Semaan Faculty of Engineering and Architecture, American University of Beirut, Beirut, Lebanon; c College of Medicine and Health Sciences, Dept of Public Health and Epidemiology, Khalifa University, Abu Dhabi, United Arab Emirates; d Harvard T. H. Chan School of Public Health, Boston, MA, USA; e College of Computing and Mathematical Sciences, Dept. of Computer Science, Khalifa University, Abu Dhabi, United Arab Emirates; f Center for Cyber-Physical Systems, Khalifa University, Abu Dhabi, United Arab Emirates; g Center for Interdisciplinary Digital Sciences (CIDS), Department Information Services and High Performance Computing (ZIH), TUD Dresden University of Technology, Dresden, Germany; h College of Computing and Mathematical Sciences, Dept of Mathematics, Abu Dhabi, United Arab Emirates; i IBM, New York, NY, USA

**Keywords:** Entropy, Mutual information, Information theory, Genetic distance, Genetic maps

## Abstract

Principal Component Analysis (PCA) is a powerful multivariate tool allowing the projection of data in low-dimensional representations. Nevertheless, datapoint distances on these low-dimensional projections are challenging to interpret. Here, we propose a computationally simple heuristic to transform a map based on standard PCA (when the variables are asymptotically Gaussian) into an entropy-based map where distances are based on mutual information (MI). Moreover, we show that in certain instances our proposed scaled PCA can improve cluster identification. Rescaling principal component-based distances using MI results in a representation of relative statistical associations when, as in genetics, it is applied on bit measurements between individuals' genomic mutual information. This entropy-rescaled PCA, while preserving order relationships (along a dimension), quantifies relative distances into information units, such as “bits”. We illustrate the effect of this rescaling using genomics data derived from world populations and describe how the interpretation of results is impacted.

## 1 Introduction: the problem of correlation

Correlation between two variables *X* and *Y* does not adequately reflect the information distance between them even when assuming that both variables are normally distributed. This also applies in the class of rapid convergence to the normal, or “thin tailed” distributions that result from approximation behavior [1]. The use of squared correlation does not solve this problem either. This distortion becomes acute with Principal Component Analysis (PCA), and across the genetic two-dimensional maps, where there is a built-in correlation component.

**Fig. 1:**
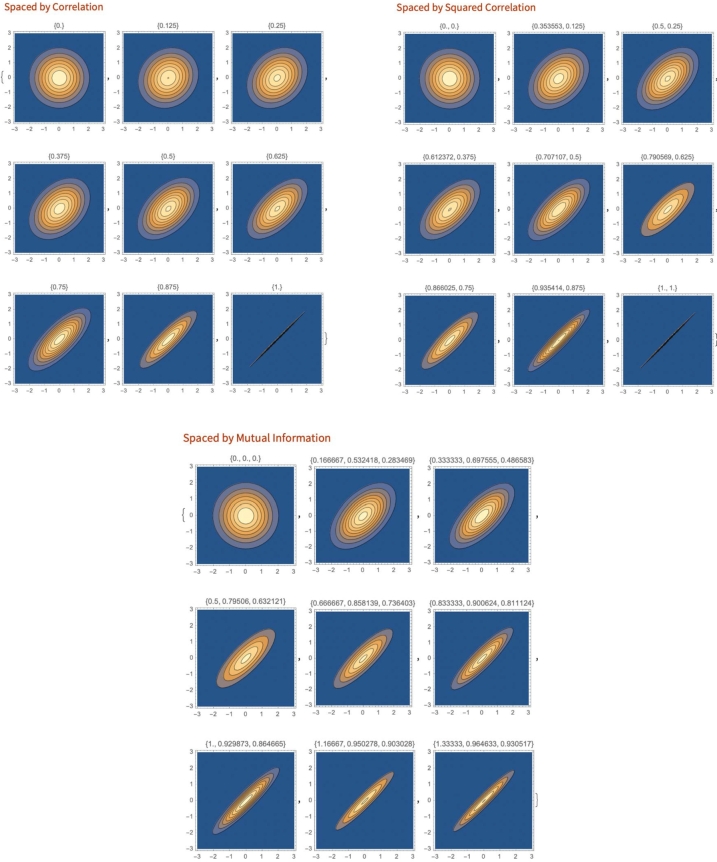
The visual intuition for the three possible methods for informational distances. We generate bivariate normal distributions for *X* and *Y*, and represent the iso-densities on the *X* and *Y* axes. Each square is equidistant with respect to the parameters 1) correlation, *ρ* (top left), 2) correlation squared (top right), *ρ*^2^, and 3) Mutual Information (bottom center), MI to the one to its left and its right, above and below it, as well as on the diagonal. The parameters in brackets are {*ρ*} for the top left, {*ρ*,*ρ*^2^} for the top right, and {*MI*, *ρ*, *ρ*^2^} for the bottom center. The square of the correlation was selected because it maps to the explained variance in traditional regression analyses. MI seems to match the visual representation of associated randomness.

For instance, when correlating 2 vectors $X_{1}$ and $X_{2}$ against *Y* (assuming it is the basis), the information does not scale linearly (even though correlation reflects a measure of the noise in a linear dependence). Hence, there is a need for some scaling of the correlation metric. For example, a 0.5 correlation is vastly –and disproportionally– inferior to 0.7; Fig. 1.

### 1.1 Information and correlation

**Fig. 2:**
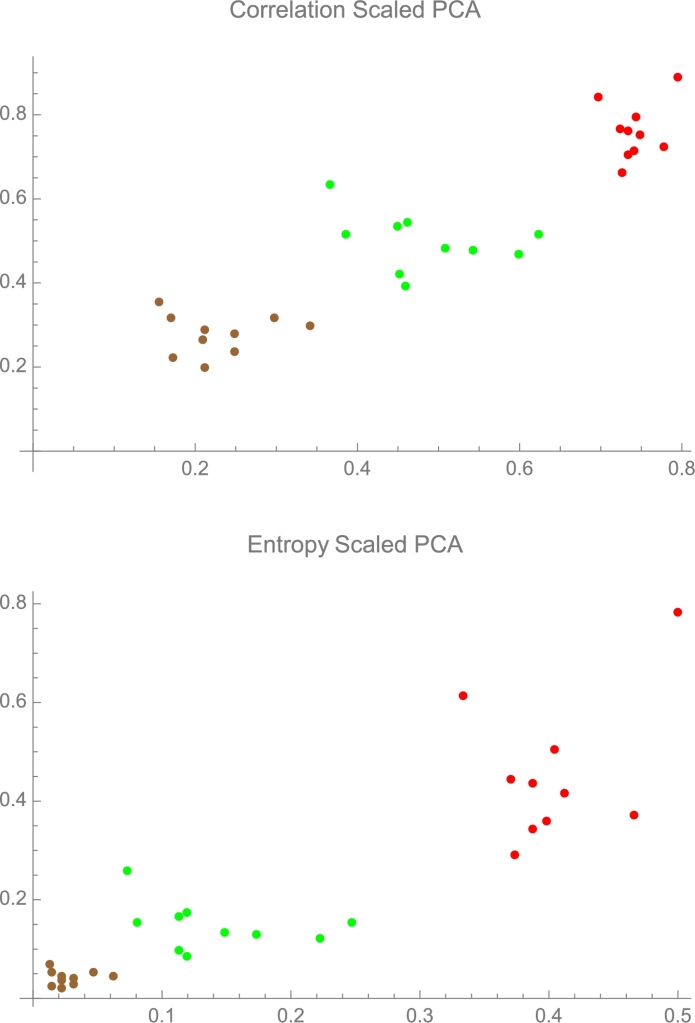
A theoretical example showing how entropy-rescaled principal components (PCs) change the relative distances to make them linear to information. This is made possible due to the information-theoretic optimality of the PCs under thin-tailed distributions. The model illustrates how ordinal relationships are conserved on each dimension, under the transformation axis-wise, but the cardinal distances are significantly altered.

**Fig. 3:**
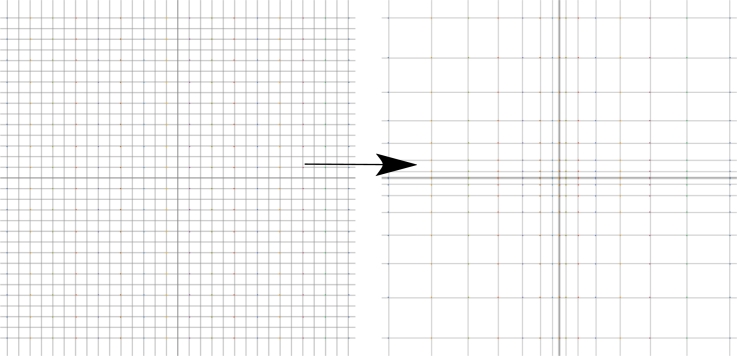
Transformation of PCA maps to accommodate informational distances. Since the maps are built by positioning the correlation (or covariance) with respect to Principal Component *PC*_*n*_ and *PC*_*m*_, *m* > *n* > =1 on the *x* and *y* axes respectively, our correction corresponds to multiplying the values of the axes by $-\text{sgn}(\rho )\frac{1}{2}\log\left(1-\rho^{2}\right)$, which is visually equivalent to stretching the map along both the *x* and *y* axes.

It has been demonstrated that metrics of non-linearity can be misleading, hence the need to “linearize” the type of metric that is used. Perceptual constraints are compounded by the non-linearity of the measure; for instance, Soyer et al. [2] showed that the real inferential and practical implications are almost always overlooked, even by the most experts in the field and all interpretation errors go in one direction, the *fooled by randomness* one (i.e., underestimation of noise) [3]. That 70% of econometricians misinterpreted their own results is quite telling. Goldstein and Taleb [4] documented a version of the effect showing that professionals and graduate students alike erroneously interpret mean deviation as standard deviation, therefore underestimating volatility, especially under non-normality. It has been shown in [5] that correlation is not additive across subsections of the domain under consideration – even when the variables are Gaussian. There are inherent limitations that are further exacerbated by the specificity and scaling of the correlation metric. A 0.5 correlation is significantly weaker than a 0.7 correlation, providing much less information—specifically about 5/7 as much. In fact, a 0.5 correlation conveys only between 0.06 and 0.14 of the information, assuming a 1 correlation has an information content of 1 and a 0 correlation has none. Clearly, it is erroneous to evaluate correlation without rescaling to allow for a relative interpretation. To facilitate better comparison, a more rigorous rescaling method is needed, a method that avoids presenting nonlinear measures, as the one proposed here. Entropy methods being additive (unlike correlation) can solve the problem, see Fig. 2 and Fig. 3; the former shows an example for rescaling of synthetic data, and the latter illustrates how transformation of 2D PCA maps can accommodate informational distances.

Not all branches of research are misled by correlation as a relatively uninformational metric. Machine learning loss functions rely on cross-entropy methods [6]. Since DNA is, certainly, *information*, an information-theoretic metric would be most preferable to any other standard metric in current use [7].

Further, since mutual information maps to “how much should one dynamically gamble on *X* knowing *Y*”, its information-theoretic quality is most applicable to genetic distance. Further, in addition to PCA analysis, entropy methods are helpful to properly scale runs of homozygosity (ROH) (that is, contiguous lengths of homozygous genotypes that are present in an individual due to parents transmitting identical haplotypes to their offspring). These are attributes that emerge naturally in phylogenetic situations that PCA has been used to sort out [8], [9].

Some analyses have been performed to explain how information is organized, as in acoustic signals, by applying PCA to mutual information matrices [10]. Similarly, information theory-based genetic distances (Mahalanobis distance) have been successfully applied to measure phenotypic relatedness [11]. These approaches are distinct from the applications considered here. Information measures are applied to PCA components, noting that they are, in their own right, measures of correlation themselves. This addresses a distinct feature of how components encode information.

Other criticisms of PCA have been recently made: PCA has an array of weaknesses, many of which are related to sample size mismatch, ability to perform cherry picking, or the insufficiency of representation in two dimensions, as reported in [12]. However, these are standard statistical problems that exist because of flaws in the applications rather than a fundamental structural problem, and fixable with more rigorous but standard checks. Our approach shows aforementioned fundamental PCA issues are not curable by these conventional checks.

For population genetics, the information-scaled component ties the expected number of segregating sites and not to the number of lineages. It involves a harmonic sum $4N_{e}\mu \sum_{k\leq N}1/k$ where $N_{e}$ is effective population size, and *μ* is the mutation rate [8]. The larger the effective size of the population, the larger is the number of segregating sites, reflecting greater genetic diversity within the population.

## 2 Methods

First, we propose a new approach to map PCs using mutual information based on the following convenient property. Because PCA vectors for Gaussian variables are orthogonal both for correlation and mutual information, a simple heuristic can be applied for the translation. Second, the precise mathematics applied to genetics are expressed in matrix form, mapping to their exact implementation on population maps. Finally, using full genomic data, we show the results as applied to the world population, as well as a subsample of it, with comments on the significant divergences between methods. Proofs and derivations are offered to clarify nomenclature as well as to provide details on normalizations and other relevant analyses.

### 2.1 PCA under mutual information

We observe that conventional Principal Component Analysis proposes distances between groups and variables based on representation on maps built as follows.

Let $\left(X_{1},\ldots ,X_{n}\right)$ be the original vectors (in $R^{m}$), and $\left(\pi_{1},\ldots ,\pi_{n}\right)$ the orthogonal principal components ordered by decreasing variance (details of computation follow in Equations (8) through (13)). Two–dimensional principal component representation typically maps $X_{i}$ in Cartesian coordinates according to a metric *μ* such that the coordinates become

$$d_{i}=\left(\mu (X_{i},\pi_{j}),\mu (X_{i},\pi_{j^{\prime }})\right)$$

where typically $j^{\prime }=j+1$. The same logic applies to three dimensions.

The function μ(.) in common use is expressed by the dot product $<X_{i},\pi_{j}>$ scaled by $\frac{1}{n-1}$, or its decomposition via the scaled correlation

(1) $$\mu (X_{i},\pi_{j})=\rho_{X_{i},\pi_{j}}\sigma_{X_{i}}\sigma_{\pi_{j}}$$

where $\sigma_{U}^{2}=\text{var}(U)$, and $\rho_{U,V}=\frac{\text{cov}(U,V)}{\sigma_{U}\sigma_{V}}$, and when the *X* are normalized,

(2) $$\mu (X_{i},\pi_{j})=\rho_{X_{i},\pi_{j}}\sqrt{\lambda_{j}}$$

where $\lambda_{j}$ is the eigenvalue associated with the principal component $\pi_{j}$.

We will revisit with a matrix notation expressing the suggested transformations as applied to genetic analysis.

### 2.2 Mutual information

As per the standard definition in the literature [13], $I_{X,Y}$ the mutual information between random variables *X* and *Y* reads:

(3) $$I_{X,Y}=\int_{D_{X}}\int_{D_{Y}}f(x,y)\log\left(\frac{f(x,y)}{f(x)f(y)}\right)\;dx\;dy$$

and

$$\log\frac{f(x,y)}{f(x)f(y)}=\log\frac{f(x|y)}{f(x)}=\log\frac{f(y|x)}{f(y)}.$$

In effect, and what is relevant to genetics, mutual information is the Kullback-Leibler divergence between two distributions: the joint distribution f(x,y) and the product f(x)f(y) evaluated with respect to the joint distribution [13].

We note some difficulties translating direct frequencies into continuous functions, but in our case, the problem is solved via the property that for Gaussian distributions, independence implies zero correlation (and vice versa), allowing the transfer to MI from the pairwise correlation. (We note that common practice consists of smoothing the kernel distribution, then computing the mutual information.)

It is central that, under bivariate normality, the orthogonal principal components satisfy, for i,j≤m

(4) $$I_{\pi_{i},\pi_{j\neq i}}=0.$$

This holds for bivariate normal distributions [14], [15] (though not all distributions in the elliptical class), where uncorrelated indicates independence. Let Σ be the covariance matrix for X,Y∼N(M,Σ) where *M* is a vector of means and $\Sigma =\left(\begin{array}{cc} \sigma_{1}^{2}\; & \rho \sigma_{1}\sigma_{2}\\ \rho \sigma_{1}\sigma_{2}\; & \sigma_{2}^{2} \end{array}\right)$. Assume M=(0,0) with no loss of generality. The PDFs are $f(x)=\frac{e^{-\frac{x^{2}}{2\sigma_{1}^{2}}}}{\sqrt{2\pi }\sigma_{1}}$; the joint PDF becomes

$f(x,y)=\frac{\exp\left(-\frac{\sigma_{2}^{2}x^{2}-2\rho \sigma_{2}\sigma_{1}xy+\sigma_{1}^{2}y^{2}}{2\left(1-\rho^{2}\right)\sigma_{1}^{2}\sigma_{2}^{2}}\right)}{2\pi \sigma_{1}\sigma_{2}\sqrt{\left(1-\rho^{2}\right)}}$. So the parametrization ρ=0 implies the identity f(x,y)=f(x)f(y), namely that lack of correlation implies independence, hence absence of mutual information between *X* and *Y*, that is, $I_{X,Y}=0$.

Taking, for example, other elliptical distributions frequently used in social science, the bivariate Student t or Cauchy, ρ=0 does not indicate independence [1]. For instance, for X,Y∼ Multivariate Student t (α,ρ), the mutual information $I_{X,Y}(\alpha )$:

(5) $$I_{X,Y}(\alpha )=-\frac{1}{2}\log\left(1-\rho^{2}\right)+\lambda_{\alpha }$$

where $\lambda_{\alpha }=-\frac{2}{\alpha }+\log(\alpha )+2\pi (\alpha +1)\csc(\pi \alpha )+2\log\left(B\left(\frac{\alpha }{2},\frac{1}{2}\right)\right)-(\alpha +1)H_{-\frac{\alpha }{2}}+(\alpha +1)H_{-\frac{\alpha }{2}-\frac{1}{2}}-1-\log(2\pi )$, where csc⁡(.) is the cosecant of the argument, B(.,.) is the beta function, and $H{\left(.\right)}^{\left(r\right)}$ is the harmonic number $H_{n}^{r}=\sum_{i=1}^{n}\frac{1}{i^{r}}$ with $H_{n}=H_{n}^{\left(1\right)}$. We note that for $\lambda_{\alpha }\underset{\alpha \to \infty }{\to }0$, the limit of the mutual information (5) corresponds to the Gaussian case.

This makes the proposed transformation heuristic more straightforward than alternatives to PCA such as the t-distributed stochastic neighbor embedding (t-SNE) method. We also note that the (standard) original stochastic neighbor embedding technique does not reflect information-theoretic distances; its aim is to reduce dimensionality.

We also note Linsker's results [16] showing that the conventional PCA provides an information-theoretic optimality in the context of understanding how neural networks perceive features as important, and show that this optimizes entropy. Much of that formalism is echoed when applying the fluctuation dissipation theorem to Linsker response and Onsager's equations [17], [18]. Together, these provide contexts ranging from applications to mapping neuronal responses in brains, to modern neural network-based AI applications, in biological pathways analyses, and other applications.

Additivity: We note that $I_{X,Y}$ is additive across partitions of $D_{X}$ and $D_{Y}$, since $I_{X,Y}=E\left(\log f(x,y)\right)-E\left(\log f(x)\right)-E\left(\log f(y)\right)$. Consider the additivity of measures on subintervals $\int_{A\cup B}f\;d\mu =\int_{A}f\;d\mu +\int_{B}f\;d\mu$.

### 2.3 Re-scaling PCA distances using mutual information

We note that irrespective of parametrization of *X* and *Y*, when the distributions are jointly Gaussian with $\rho_{X,Y}$, $I_{X,Y}=-\frac{1}{2}\log\left(1-\rho^{2}\right)$.

$I_{X,Y}$ the mutual information between random variables *X* and *Y* and joint PDF f(.,.), because of its additive properties, allows a representation of relative associations, via the re-scaling function

(6) $$r_{X,Y}=-\text{sgn}(\rho_{X,Y})\frac{1}{2}\log\left(1-\rho_{X,Y}^{2}\right).$$

We use the signed correlation to show the direction of the information: MI shows strength of association, not its direction, and for a monotonic linear function, the association is preserved in the negative domain. Hence, Eq. (2) can be modified for rescaling (marked as $\mu^{\prime }$)

(7) $$\mu {\left(X_{i},\pi_{j}\right)}^{\prime }=-\text{sgn}(\rho_{X_{i},\pi_{j}})\frac{1}{2}\log\left(1-\rho_{X_{i},\pi_{j}}^{2}\right)\sqrt{\lambda_{i}},$$

as shown in Fig. 2 (PCA of synthetic data) and Fig. 3.

### 2.4 In matrix notation

Using matrix notation (mapping to our implementation), the problem is expressed as follows: For PCA analysis of genetic variants, normalizations follow some domain-specific conventions [19]. For this reason, a notation for single nucleotide polymorphism (SNP) data is adapted, labeling the data *g*, for genetic, representing the general variates *X* in the above discussion. Centering and scaling in the correct order yields a correlation matrix, as follows. We start by defining a matrix $G=(g_{ij})$ with features indexed by $i\in Z_{m}$ samples, and $j\in Z_{n}$. These features could be biallelic diploid SNPs coded in $Z_{2}$ (**G** corresponds to matrix *C* in [20]). In this case, it is common to assign values of 0 to the major haploid allele, 1 to the minor haploid allele. Diploid alleles are then coded as 0 for homozygous major alleles, 1 for heterozygous alleles, and 2 for homozygous minor alleles. This additive encoding scheme is the de facto standard for Principal Component Analysis on genetic data (SmartPCA) [19]. Such datasets have usually been filtered to remove SNPs with very rare minor alleles and with large Hardy-Weinberg deviations.

Note that the centering by rows for genotypic analysis differs from Patterson et al. [20], but conforms with Price et al. [19]; SmartPCA computes the appropriate correlations with “altnormstyle: NO”.

Define

(8) $$m_{i}=\frac{1}{n-1}\sum_{j\in Z_{n}}g_{ij},$$

(9) $$\sigma_{ii^{\prime }}=\frac{1}{n-1}\sum_{j\in Z_{n}}(g_{ij}-m_{i})(g_{i^{\prime }j}-m_{i^{\prime }}),$$

(10) $$\sigma_{i}^{2}=\sigma_{ii},$$

(11) $$Z=\left(\frac{g_{ij}-m_{i}}{\sigma_{i}}\right)$$

(12) $$\rho_{ii^{\prime }}=\frac{\sigma_{ii^{\prime }}}{\sigma_{i}\sigma_{i^{\prime }}}.$$

Then $ZZ^{T}=(n-1)\left(\rho_{ii^{\prime }}\right)$.

Accordingly, $ZZ^{T}=\left(\sum_{j\in Z_{n}}\frac{\left(g_{ij}-\mu_{i})(g_{i^{\prime }j}-\mu_{i^{\prime }}\right)}{\sigma_{i}\sigma_{i^{\prime }}}\right)=(n-1)\left(\frac{\sigma_{ii^{\prime }}}{\sigma_{i}\sigma_{i^{\prime }}}\right)=(n-1)\left(\rho_{ii^{\prime }}\right)$. Therefore, the correlation matrix **C** may be represented by $C=(n-1)ZZ^{T}$.

(13) $$C=\left(\rho_{ii^{\prime }}\right)=\frac{1}{n-1}ZZ^{T}=\text{cov}(Z,Z^{T}).$$

**C** is symmetric and positive definite. Since, for any vector **w**, the expression $w^{T}\mathrm{Cw}=\frac{1}{n-1}{\left(Z^{T}w\right)}^{T}(Z^{T}w)\geq 0$, it follows that **C** is positive definite. Also, $C^{T}=\frac{1}{n-1}{\left(ZZ^{T}\right)}^{T}=\frac{1}{n-1}ZZ^{T}=C$, and so is symmetric.

The diagonalization of **C** provides a decomposition of the feature vectors into an orthogonal set that spans the subspace containing the samples. The $U^{T}Z$ rows are orthogonal, and the covariance diagonal.

Given that **C** is positive definite and symmetric, **C** is diagonalized by an orthonormal matrix **U** of the normalized orthogonal eigenvectors to yield a diagonal matrix **D**, so that CU=UD. $S^{2}=(n-1)D$ is in common usage so that $(ZZ^{T})U=US^{2}$. Therefore, $D=U^{T}\mathrm{CU}=\text{cov}((U^{T}Z),{\left(U^{T}Z\right)}^{T})=\frac{1}{n-1}U^{T}ZZ^{T}U$. Since **D** is diagonal, the $U^{T}Z$ rows are orthogonal, and the covariance **D** in that basis is diagonal.

We can identify the *n* columns, *m* rows, matrix of *n* feature-wise orthogonal principal components $\pi_{i}$ as:

(14) $$P=U^{T}Z$$

Note that, since the covariances of **P**, $\text{cov}(P,P^{T})=D$ are diagonal, the rows are orthogonal, as noted previously. The matrix

(15) $$V={\left(n-1\right)}^{-1/2}D^{-1/2}P=S^{-1}U^{T}Z$$

is normalized so that $VV^{T}=I$. **V** is half-orthonormal; the transposes are not: $V^{T}V\neq I$. The reason for this is that the number of individual vectors of SNPs for the individuals in **Z** does not span the space of SNP vectors since m≪n. These are the familiar matrices in the singular value decomposition commonly used in population genetics [20], [19]

(16) $$Z=USV^{T}.$$

This decomposition also shows that the vectors in $V^{T}$ represent an orthogonal basis in which **Z** can be represented, and so covers the subspace spanned by **Z**.

Also, $\text{cov}(S,S^{T})=U^{T}\text{cov}(Z,Z^{T})U$ will be useful.

We define the correlation matrix

(17) $$M=\text{cor}(Z,P^{T})$$

Then

(18) M=U

$M=[\text{cov}(Z,{Z^{T})]}^{-1/2}\text{cov}(Z,P^{T})[\text{cov}(P,{P^{T})]}^{-1/2}$. Noting that

$$\text{cov}(Z,Z^{T})=\frac{1}{n-1}U^{T}S^{2}U$$

$$\text{cov}(P,P^{T})=\frac{1}{n-1}S^{2}$$

and

$$\text{cov}(Z,P^{T})=\frac{1}{n-1}ZZ^{T}U=\frac{1}{n-1}US^{2}$$

Then

$$M=US^{-1}U^{T}US^{2}S^{-1}=U.$$

This is therefore the standard principal component matrix that we expect, *and*, since this is a correlation, this may be re-scaled as mutual information. The information re-scaled version $M^{\prime }$ becomes

$$M^{\prime }=R(M)=R(U),$$

where *R* is the matrix whose entries are computed according to Equation (6).

## 3 Application to genetic distance

**Fig. 4:**
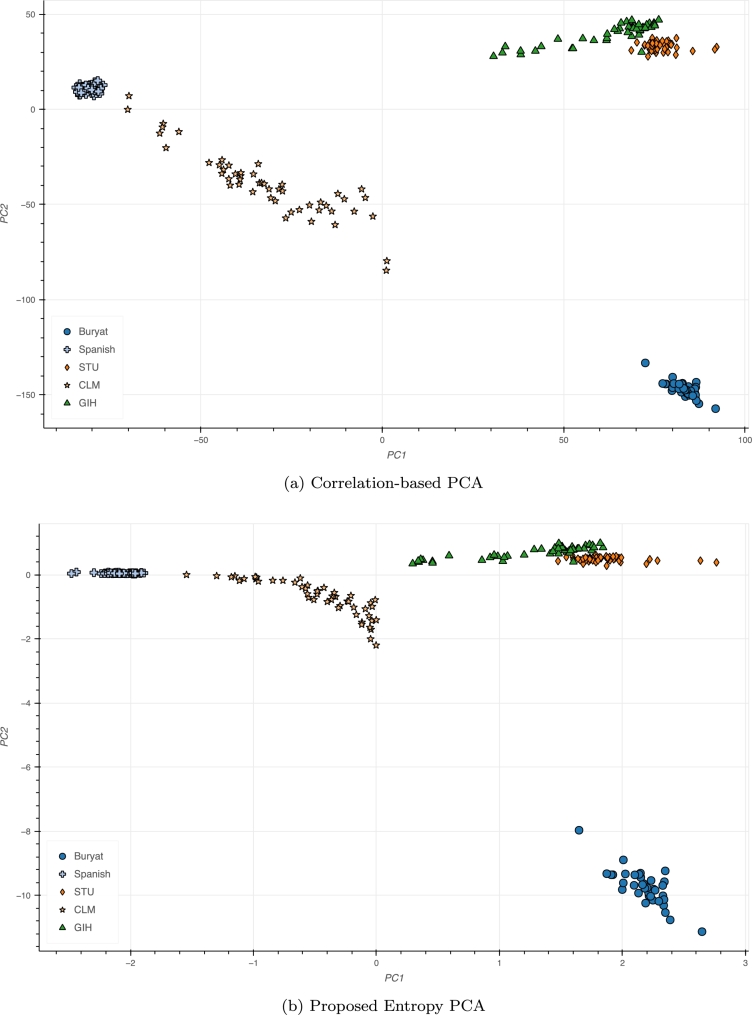
Conventional Principal Component Analysis for 5 populations: Buryat, Spanish, Sri Lankan Tamil in the UK (STU), Colombian in Medellín, Colombia (CLM) and Gujarati Indians in Houston, Texas, USA (GIH). While the gap between CLM and GIH appears rather large in conventional PCA, comparable to the distance between CLM and Buryat, rescaling places CLM substantially closer to GIH, shown in (b).

We investigate the visualization of genetic distances in world populations. To this end, we select contemporary populations from the Allen Ancient DNA Resource (AADR) Human Origin dataset version 54.1, which in turn comprises samples from the 1000 Genomes Project [21]. The following populations were selected for constructing the Principal Component Analysis shown in Fig. 4: Columbians from Medellin (CLM, 94 samples), Buryat from Russia (37 samples), Tamils sampled from United Kingdom (STU, 98), Gujarati India (GIH, 102) and Spanish (172). The microarray dataset contains 597,573 typed loci. The total genotyping rate is 0.999255.

**Fig. 5:**
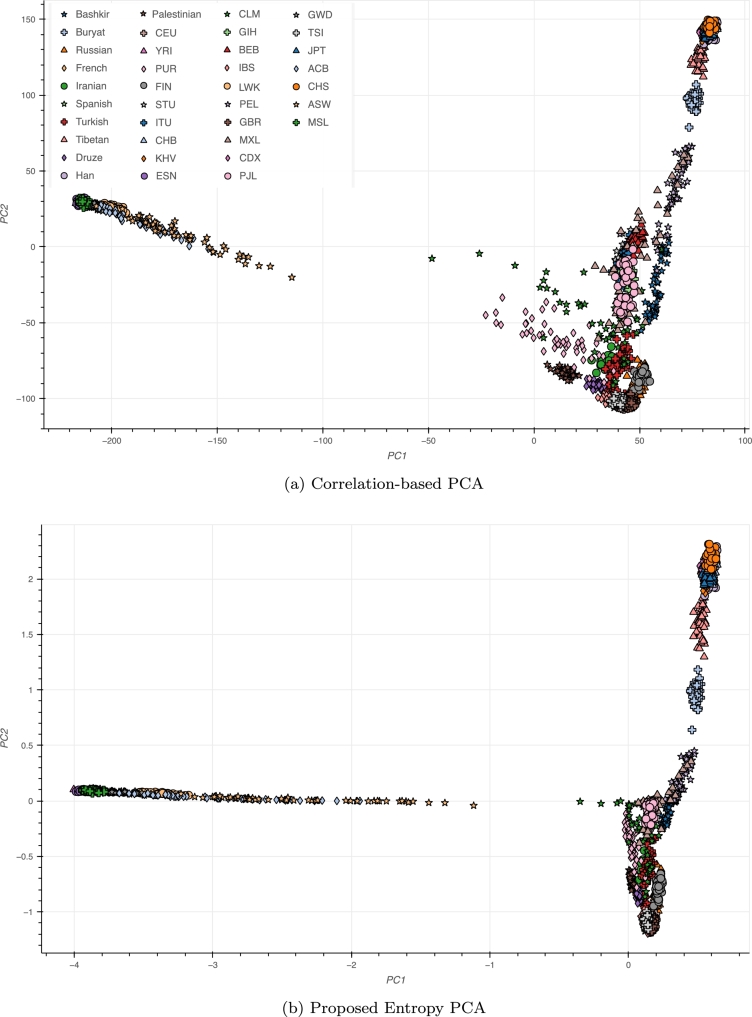
A different world view: the commonly observed triangular PCA shape of world populations undergoes proximity rearrangements using information-based rescaling. Non-African and non-Asian populations are much closer together in (b).

Fig. 5 provides a new perspective on the common PCA plot of world populations derived from the 1000 Genomes Project (all used populations are listed in Supplementary Material).

The harmonic series character of the scaling of segregating sites, which approximately scales logarithmically, provides gives a picture of how many branches, marked by discriminating SNPs, separate population structures in the data. The information mapping therefore captures a qualitative picture of how many surviving branching events separate populations, rather than the time between branches. Hence, the time reflected in early, long-legged branch edges is lost (Fig. 3). Therefore, branching events to a most recent common ancestor, rather than time to a most recent common ancestor, are observed and counted (Fig. 4, Fig. 5).

While the Asian and African clines still determine the overall structure (though nearer to the axes), the remaining world populations are closer together. For example, for Puerto Ricans (PUR) and Colombians (CLM), conventional PCA spreads them along the beginning of the African cline, whereas rescaling shows them in the vicinity of other Latin American populations (Mexicans and Peruvians). Iranians, Turkish, Palestinian, Druze, French, Iberian (IBS), British (GBR), Russian, Finnish, Puerto Rican, and the majority of Colombians all form a much tighter cluster in the rescaled PCA, indicating that these populations are not as far from each other as the conventional PCA suggests.

As with PCA in general, the approach is qualitative. PCA's estimate of genetic distance is rough, but the projection can be informative of similarities between populations as well as time of separation; in this scaling, the mapping provides a qualitative measure of branching events. A shared weakness is that this does not offer a statistical test that contrasts relationships, time, or branching events.

### 3.1 Importance of the MI scaled PCA in genomics

The use of information theoretic quantities is not novel in genomics [22], [23], [24]. In the following, we identify two main advantages for using the MI scaled PCA:

(i) **Improved interpretability:** Within the scaled PCA, the distance between different genetic elements can be quantified in terms of bits, nats and other information units. This quantification renders the results more reproducible and allows for better comparative analyses from various sources. Quantifying differences between populations or clusters can lead to a better characterization of their evolutionary lineages and the genomic regions that contribute to the observed population structure.

(ii) **Nonlinear relationships:** Mutual information is a much improved method in dissecting nonlinear associations [13]. In particular, using MI as a distance measure in genomics can be an effective approach for understanding dependencies and relationships between genetic elements, i.e., comparing gene expression levels with SNP variations or correlating a specific peptide with variations in gene expression. Unlike traditional Euclidean distance or correlation-based methods, MI can capture both linear and nonlinear dependencies between variables, making it especially useful in genomic data analysis, especially in cases of admixture where different populations have interbred over many generations.

(iii) **Improved clustering:** In terms of clustering, it can produce more pronounced cluster distances, in particular when their centers of masses are highly correlated with the projected reduced-dimension plane. To illustrate our argument, let us assume a *m*-dimensional system and the center of mass of two clusters $x_{1},\;x_{2}\in R^{m}$. Then we apply PCA and reduce the dimensionality of the problem to a single dimension i.e., the principal component $\pi_{1}$. The center of masses of the clusters will be projected on the first component as $\mu_{i}=\rho_{\pi_{1},x_{i}}\sqrt{\lambda_{i}}\in R$, i=1,2, and the corresponding distance (discriminant function) will read $\Delta \mu =\frac{\left|\mu_{2}-\mu_{1}\right|}{\sigma }$, where $\sigma^{2}$ is the pooled variance of the two clusters. Note that the latter is the Mahalanobis distance of the two cluster centers. The MI scaled distance of the projected center of mass will read

$$\frac{\sigma^{2}}{\lambda_{1}}\Delta {\overset{˜}{\mu }}^{2}=\frac{1}{\lambda_{1}}{\left({\overset{˜}{\mu }}_{1}-{\overset{˜}{\mu }}_{2}\right)}^{2}=\frac{1}{4}{\left(\ln\frac{1-\rho_{\pi_{1},x_{2}}^{2}}{1-\rho_{\pi_{1},x_{1}}^{2}}\right)}^{2},$$

for positive correlation coefficients $\rho_{\pi_{1},x_{i}}$, i=1,2. Now we are interested in situations where clusters are not easily distinguishable, i.e. the two projected center of masses are close to each other $\rho_{\pi_{1},x_{2}}-\rho_{\pi_{1},x_{1}}\ll 1$. In this regard, we can linearize the MI scaled distance $\Delta \overset{˜}{\mu }$ of the clusters, when the corresponding correlations are close to each other, and compare it with the linear distance Δ*μ*:

(19) $$\frac{\sigma^{2}}{\lambda_{1}}(\Delta {\overset{˜}{\mu }}^{2}-\Delta \mu^{2})\approx (\frac{\rho_{\pi_{1},x_{1}}^{2}}{{\left(1-\rho_{\pi_{1},x_{1}}^{2}\right)}^{2}}-1){\left(\rho_{\pi_{1},x_{2}}-\rho_{\pi_{1},x_{1}}\right)}^{2}.$$

The latter formula is positive when the MI scaled distance is larger from the linear one for close enough correlation coefficients. In particular, this parabola fits the saddle of the original double-well $\Delta {\overset{˜}{\mu }}^{2}-\Delta \mu^{2}$ function. It is interesting to observe that for high enough correlation coefficients, larger than 0.61, the MI scaled distance provides more pronounced cluster distances than the linear PCA one i.e., $\Delta {\overset{˜}{\mu }}^{2}>\Delta \mu^{2}$. An one-tailed t-test can be used to statistically check if the correlation of the cluster center of mass to the principle component is larger than the above threshold value. A multivariate extension of this test is the Hotteling's $T^{2}$ test.

At this point, it is natural to compare scaled PCA versus nonlinear dimensionality reduction (DR) methods, such as tSNE or UMAP. The nonlinear nature of the latter allows them to go beyond the limitations of correlation in a manner similar to that of scaled PCA. Moreover, nonlinear DR methods typically allow for discriminating data clusters better that traditional PCA and potentially better than the scaled PCA. However these nonlinear DR methods do not allow for interpretable distances, as our proposed MI scaled PCA. The following table summarizes the differences between PCA, scaled PCA and nonlinear DR methods. (See Table 1.)

**Table 1:** Comparison of PCA, MI scaled PCA, and Nonlinear dimensionality methods (DR). The H, M, L account for high, medium and low, respectively.

	Interpretability	Nonlinear relationships	Clustering
PCA	L	L	L
Scaled PCA	H	H	M
Nonlinear DR	L	H	H

### 3.2 Discussion and conclusion

To conclude, we show how, under conditions satisfied in population genetics, to efficiently and effectively convert a principal components-based map to one representing information-based distance. Using the methodology in [12], there are more than 200,000 published results that may be affected by this simple change of metric, with conclusions that would need to be reevaluated.

The proposed scaled PCA is intended to provide an alternative output and interpretations for the organization of phylogeographic information revealed by PCA. Such scaling emphasizes the number and effect of lineages surviving coalescence. The result, contrasting in scale between lineage weighted and time weighted scales as in neural homunculi, aids in graphically recognizing features enabled by newer technologies and data acquisitions. This aids in the interpretability of genetic distances by quantifying them in information units that potentially enhances clustering separation by segregating markers, providing contrast with distances that scale with time more than by lineage counts. The analysis shown in Fig. 2 demonstrates, using a theoretical example, that the almost entire variance on the mutual information PCA matrix can be captured by the first two principal components, highlighting a more efficient dimensionality reduction compared to the correlation matrix. Mutual information, accounting for non-linear dependencies, may provide a more comprehensive representation of underlying data structures. It provides a more robust and informative approach for understanding the dependencies between variables compared to traditional Euclidean-based methods in the identification of genetic structures (population clusters). While there are widely used dimensionality reduction methods such as t-SNE or UMAP which outperform linear PCA in terms of clustering resolution. The MI scaled PCA methods presented here, due to their non-linear nature, offer quantifiable measures that better distinguish evolutionary features and improve results interpretation.

We also note that there are other opportunities that could benefit from the approach presented here that have not been explored in this study. Linsker [16] argues how in conventional PCA neural networks perceive features as important in terms of information optimization. Through information optimization, many of the same structures appear in the fluctuation dissipation theorem, and in Onsager's equations and reciprocity theorem [17], [18]. Further, this application can be used to map neuronal responses in biological brain tissues, and in subsequent development of neural networks applied to computational artificial intelligence problems. These typically inherit many of the same measurements. Finally, this approach can be used in non-equilibrium thermodynamics applications for the analysis of biological pathways, to help in understanding the emergence of regulatory self-organization mechanisms. In particular, it can be applied in the problem of cell decision making in multicellular system and embedded to recent information-based theories [25].

While we highlight the applications of our approach in population genetics and potentially in other fields, these applications have not been exhaustively tested within the scope of this research. For instance, biomarker identification is a prominent field of application [26], [27]. We limited our paper to two examples, one of which used a comprehensive dataset representing many populations throughout the globe. Future research will show how divergent our results are from those obtained using current PCA methods.

## CRediT authorship contribution statement

**Nassim Nicholas Taleb:** Writing – original draft, Conceptualization. **Pierre Zalloua:** Writing – review & editing, Methodology. **Khaled Elbassioni:** Validation, Methodology. **Haralampos Hatzikirou:** Writing – review & editing, Validation, Methodology. **Andreas Henschel:** Writing – review & editing, Methodology, Formal analysis. **Daniel E. Platt:** Writing – review & editing, Methodology, Formal analysis, Conceptualization.

## Declaration of Competing Interest

The authors declare no conflict of interest.

## Appendix A Supplementary material

Supplementary Material List of world populations used to derive the PCAs in Figures 4 and 5. Supplementary Material
