# Supplementary material for: Informational rescaling of PCA maps with application to genetic distance
Source: Comput Struct Biotechnol J. 2024 Dec 11;27:48–56. doi: 10.1016/j.csbj.2024.11.042 (PMC11719279; doi:10.1016/j.csbj.2024.11.042)
Supplement: Supplementary Material — List of world populations used to derive the PCAs in Figures 4 and 5. [file mmc1.pdf]

A. Supplementary Material

The following populations were taken from the AADR set (exact population labels as in AADR are used):

Samples	Populations	Datasets	Populations
92	ACB.DG	102	ITU.DG
55	ASW.DG	38	Iranian.HO
85	BEB.DG	104	JPT.DG
53	Bashkir.HO	95	KHV.DG
37	Buryat.HO	99	LWK.DG
93	CDX.DG	85	MSL.DG
99	CEU.DG	62	MXL.DG
103	CHB.DG	68	PEL.DG
103	CHS.DG	96	PJL.DG
94	CLM.DG	99	PUR.DG
41	Druze.DG	38	Palestinian.DG
99	ESN.DG	71	Russian.HO
97	FIN.DG	98	STU.DG
61	French.HO	172	Spanish.HO
90	GBR.DG	107	TSI.DG
102	GIH.DG	97	Tibetan.HO
112	GWD.DG	50	Turkish.HO
45	Han.DG	101	YRI.DG
103	IBS.DG		
